# Fear of cancer recurrence in oncohematological patients: assessment instruments and evidence-based psychological interventions — a systematic review

**DOI:** 10.3389/fpsyg.2025.1635641

**Published:** 2025-11-19

**Authors:** Ana Sancho-Martínez, María Rueda-Extremera, Sergio Alejandre-Carmona, María Cantero-García

**Affiliations:** 1 Faculty of Psychology and Health Sciences, Distance University of Madrid (UDIMA), Madrid, Spain; 2 La Paz University Hospital, Madrid, Spain

**Keywords:** hematologic cancer, assessment, therapy, quality of life, fear of recurrence

## Abstract

**Introduction:**

Oncohematological patients undergo a complex emotional adaptation process, in which Fear of Cancer Recurrence (FCR) is one of the most prevalent concerns. This fear not only negatively impacts their psychological well-being but is also associated with a significant reduction in quality of life.

**Objective:**

This study aims to conduct a systematic review of the available evidence regarding: (1) The factors associated with FCR in oncohematological patients. (2) The validity and reliability of the instruments used for its assessment. (3) The therapeutic interventions designed to mitigate FCR in this population, with particular focus on those supported by solid empirical evidence.

**Methodology:**

A systematic review was conducted following the PRISMA guidelines. The literature search was performed in November 2024 across the PubMed, PsycINFO, Web of Science, ProQuest, SUMMON, SciELO, Redalyc, Dialnet, and Google Scholar databases. Rigorous inclusion and exclusion criteria were applied to ensure the methodological quality of the selected studies.

**Results:**

After the selection process, 11 studies meeting the eligibility criteria were included. The main findings were: (a) FCR assessment instruments: Four validated tools specifically designed for oncohematological populations were identified. (b) Therapeutic interventions: Four programs based on cognitive-behavioral therapy and third-wave therapies were found to be effective in reducing FCR in patients with leukemia, lymphoma, and multiple myeloma.

**Conclusion:**

This systematic review provides a comparative evaluation of measurement methods and the most effective interventions for addressing FCR in oncohematological patients. The findings highlight the need for further research tailored to the specific characteristics of this population, fostering the development of more precise and accessible therapeutic strategies.

## Introduction

Hematologic cancer encompasses a range of malignancies, including leukemias, lymphomas, and myelomas, representing a significant component of the global oncology landscape. According to the International Agency for Research on Cancer (IARC) of the World Health Organization (WHO), there were 20 million new cancer cases and 9.7 million cancer-related deaths in 2022. The estimated number of individuals alive 5 years post-cancer diagnosis was 53.5 million. These figures underscore the increasing global cancer burden, with projections exceeding 35 million new cases by 2050. In Spain, the Spanish Network of Cancer Registries (REDECAN) estimates that hematologic malignancies will account for 10% of all new cancer diagnoses by 2025, translating to approximately 25,770 cases, ranking behind breast, lung, prostate, and colorectal cancers. Among newly diagnosed hematologic malignancies, lymphoid-origin tumors are expected to constitute 18,357 cases, compared to 7,148 of myeloid origin. The most prevalent lymphoid neoplasms include diffuse large B-cell lymphoma (27%) and multiple myeloma (22%), whereas the most common myeloid cancers include myeloproliferative neoplasms (39%), acute myeloid leukemia (27%), and myelodysplastic syndromes (24%).

The impact of hematologic malignancies extends beyond physical consequences, exerting significant psychological distress (Chittem, 2014). One of the most prominent psychological challenges faced by cancer patients is fear, and in the context of hematologic cancer, Fear of Cancer Recurrence (FCR) has emerged as a predominant concern, representing one of the most frequently unmet needs among survivors (Barata et al., 2016; Oberoi et al., 2017; Simard et al., 2013). FCR is defined as “the fear or worry that cancer will return or spread to the same or another part of the body” (Espinoza-Salgado et al., 2023; Lebel et al., 2016). Research indicates that over half of cancer survivors experience some degree of FCR (Butow et al., 2013), which may contribute to depression and significantly impair quality of life (Koch et al., 2014). Manifestations of FCR include rumination, nervousness, and sleep disturbances, along with sadness, apathy, and irritability (Luigjes-Huizer et al., 2024). Fear of Cancer Recurrence (FCR) and Fear of Progression (FoP) represent distinct constructs. FCR refers to the fear that cancer will return after a period of remission, whereas FoP encompasses anxiety regarding disease worsening or advancement during active illness. Treating them as separate but overlapping phenomena provides greater conceptual precision and facilitates clearer interpretation of research findings (Lebel et al., 2016; Mutsaers et al., 2023).

Fear of Cancer Recurrence is intrinsically linked to patients’ experiences during and after treatment, ranking as one of the three primary predictors of distress in hematologic cancer (Raphael et al., 2020). A systematic review by Lu et al. (2023) highlighted that cancer type may contribute to variability in the association between FCR and other factors, such as social support, underscoring the necessity of investigating FCR within the unique context of each oncological process. Notably, the diagnosis and treatment of hematologic cancer are frequently associated with aggressive therapeutic regimens and prolonged adverse effects, exacerbating uncertainty about patients’ future health (Hall et al., 2018). This uncertainty often translates into a complex emotional experience in which FCR becomes an additional burden, interfering with daily life activities and adherence to routine medical follow-ups (Zhang et al., 2023). Among the ten most common unmet needs related to FCR management in hematologic cancers are the need for comprehensible information and support in addressing concerns about disease recurrence (Lobb et al., 2009). In this regard, psychoeducational interventions have proven effective in reducing FCR (Dieng et al., 2016). Recent evidence highlights that hematologic cancer survivors show particularly high levels of psychosocial distress, uncertainty, and emotional exhaustion due to the chronic and relapsing nature of these diseases. This group faces unique psychological challenges related to constant medical surveillance, treatment-induced physical changes, and fear of relapse, which contribute to persistent FCR and reduced quality of life. As noted by Shivappa et al. (2024), psychosocial distress and unmet emotional needs remain highly prevalent in this population, underlining the importance of targeted psychological interventions and adaptive coping strategies.

Addressing FCR necessitates a comprehensive understanding of its scope and implications, its impact on patients and their families, and the most effective evaluation and intervention strategies. Despite the high prevalence of FCR, no consensus has been reached regarding the most appropriate assessment instruments, which is crucial for the development of tailored, evidence-based interventions (Anderson et al., 2021). Additionally, further exploration is needed to assess the efficacy of psychological interventions designed to help patients cope with FCR. Although numerous studies have evaluated intervention effectiveness across various oncological populations (Akechi et al., 2023; Bruin et al., 2023; Dieng et al., 2016; Hall et al., 2018; Kan et al., 2021; Tauber et al., 2019; Tofthagen et al., 2025; Zhang et al., 2019) a comprehensive review specifically examining the efficacy of these approaches in hematologic cancer patients is lacking (Zhang et al., 2023). Such an analysis is essential to inform clinical decision-making and optimize patient outcomes.

Thus, the present study aims to conduct a systematic review to: (a) identify and evaluate validated instruments for assessing FCR in hematologic cancer patients, analyzing their reliability and validity, and (b) examine the most effective psychological treatments for reducing FCR in this population, specifically focusing on randomized controlled trials (RCTs) [see Table 1: PICOS Strategy (Richardson et al., 1995)].

**TABLE 1:** PICOS strategy (Richardson et al., 1995).

PICOS elements	(a) Assessment instruments	(b) Psychological treatments
Population (P)	Patients with hematologic cancer (leukemia, lymphoma, etc.).	Patients with hematologic cancer.
Intervention (I)	Assessment instruments for fear of recurrence.	Psychological treatments (e.g., cognitive-behavioral therapy, acceptance and commitment therapy).
Comparison (C)	There is no specific comparison group, but different assessment instruments can be compared.	Psychological treatments vs. standard care or no intervention.
Outcomes (O)	Validity and reliability of instruments to measure fear of recurrence.	Effectiveness of treatments in reducing fear of recurrence and improving quality of life.
Study design (S)	Validation studies, clinical trials, systematic reviews.	Clinical trials, randomized clinical trials, controlled studies (RCTs).

## Methods

This systematic review adheres to the PRISMA guidelines (Page et al., 2021). As part of the study planning, the search strategy was registered in the Open Science Framework (Sancho et al., 2024).

The literature search was conducted across multiple databases, including PubMed, PsycINFO, Web of Science, ProQuest, SUMMON, SciELO, Redalyc, Dialnet, and Google Scholar. Searches were performed in both English and Spanish, employing predefined search terms and Boolean operators (see Table 2). The search strategy used the following Boolean combinations: (“fear of cancer recurrence” OR “fear of progression”) AND (“hematologic cancer” OR “blood cancer” OR “leukemia” OR “lymphoma”). Searches were conducted in PubMed, PsycINFO, Scopus, and Web of Science. Two reviewers independently screened titles, abstracts, and full texts, and any discrepancies were resolved by discussion or consultation with a third senior author. The process followed PRISMA guidelines to ensure methodological transparency and replicability. The search process was completed on November 23, 2024. Article selection was guided by specific inclusion criteria (see Table 3).

**TABLE 2:** Search terms and Boolean operators used.

English terms	Spanish terms
“fear of recurrence,” “hematologic cancer,” “hematological malignancies,” “blood cancer,” “leukemia,” “lymphoma,” “multiple myeloma,” “fear of cancer recurrence,” “cancer recurrence anxiety,” “cancer survival anxiety,” “assessment tools,” “validated measurement instruments,” “assessment scales,” “questionnaire,” “scale,” “hematological malignancy,” “psychological treatments,” “psychotherapy,” “cognitive-behavioral therapy,” “psychological interventions,” “therapeutic approaches,” “therapy,” “treatment,” “validation,” “intervention.”	“miedo a la recurrencia,” “cáncer hematológico,” “enfermedades hematológicas,” “cáncer en la sangre,” “leucemia,” “linfoma,” “mieloma,” “miedo a la recurrencia del cáncer,” “ansiedad por la recurrencia del cáncer,” “ansiedad por la supervivencia del cáncer,” herramientas de evaluación,” “instrumentos de medida validados,” “escalas de evaluación,” cuestionario,” “escala,” “enfermedad hematológica,” “tratamientos psicológicos,” “psicoterapia,” “terapia cognitivo-conductual,” “intervenciones psicológicas,” “enfoques terapéuticos,” “terapia,” “tratamiento,” “validación,” e “intervención.”
Operadores booleanos: AND y OR	Operadores booleanos: Y y O

The following Boolean search strings and terms were adapted to the syntax of each database: (“fear of recurrence” OR “fear of cancer recurrence” OR “cancer recurrence anxiety” OR “cancer survival anxiety”) AND (“hematologic cancer” OR “hematological malignancies” OR “hematological malignancy” OR “blood cancer” OR “leukemia” OR “lymphoma” OR “multiple myeloma”) AND (“assessment tools” OR “validated measurement instruments” OR “assessment scales” OR “questionnaire” OR “scale” OR “validation”) AND (“psychological treatments” OR “psychotherapy” OR “cognitive-behavioral therapy” OR “psychological interventions” OR “therapeutic approaches” OR “therapy” OR “treatment” OR “intervention”).

**TABLE 3:** Selection criteria.

Inclusion criteria	Exclusion criteria*
- Studies published between 2010 and 2024.	- Studies in patients with types of cancer other than hematologic cancers.
- Studies conducted with patients diagnosed with hematologic cancer (leukemia, lymphoma, and multiple myeloma).	- Opinion articles, editorials, case reports, or case series studies.
- Studies that assess FCR using validated instruments. - Studies published in English or Spanish. - Randomized clinical trials and non-randomized studies.	- Studies without validated assessment instruments or without evaluating the effectiveness of treatments.

*Examples of excluded categories included: Studies evaluating fear of progression in solid cancers such as breast, lung, or colorectal cancer. Research exploring general anxiety or post-traumatic stress without an explicit FCR or FoP measure. Qualitative studies focusing on coping or adjustment experiences without standardized assessment tools. Intervention protocols or trial registrations published without outcome data.

Two independent reviewers screened all retrieved records in parallel across the three search iterations, assessing eligibility based on the predefined inclusion and exclusion criteria. Reference management software was used to ensure consistency and transparency—Mendeley Reference Manager (version 2.122.0, web) and Zotero (version 7.0, desktop) facilitated citation management and duplicate removal. Both the title/abstract and full-text screening stages were conducted independently by the two reviewers. Any discrepancies were resolved through discussion and consensus with a third senior reviewer, whose decision was final. This multi-step procedure ensured methodological rigor and minimized potential selection bias.

The selection process commenced with the identification of relevant articles within the databases, followed by a two-stage screening procedure based on the established criteria: (1) an initial filtering based on the title and abstract review and (2) a full-text assessment to determine suitability for inclusion. To ensure rigor and reliability, a triangulation process was conducted, whereby a third member of the research team reviewed discrepancies and contributed to the final selection.

**FIGURE 1:**
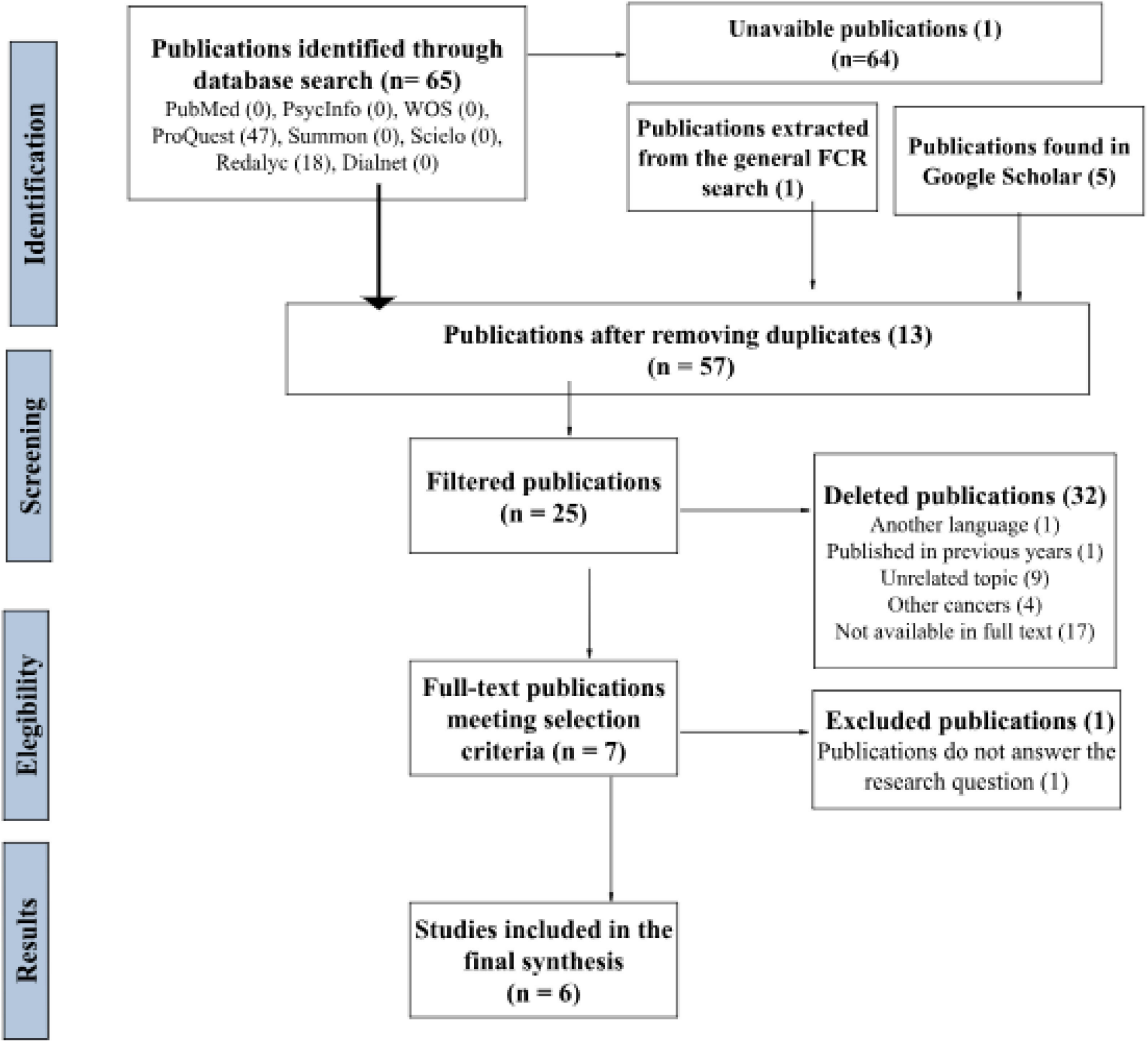
Assesment instruments. PRISMA-based systematic review process flowchart

**FIGURE 2:**
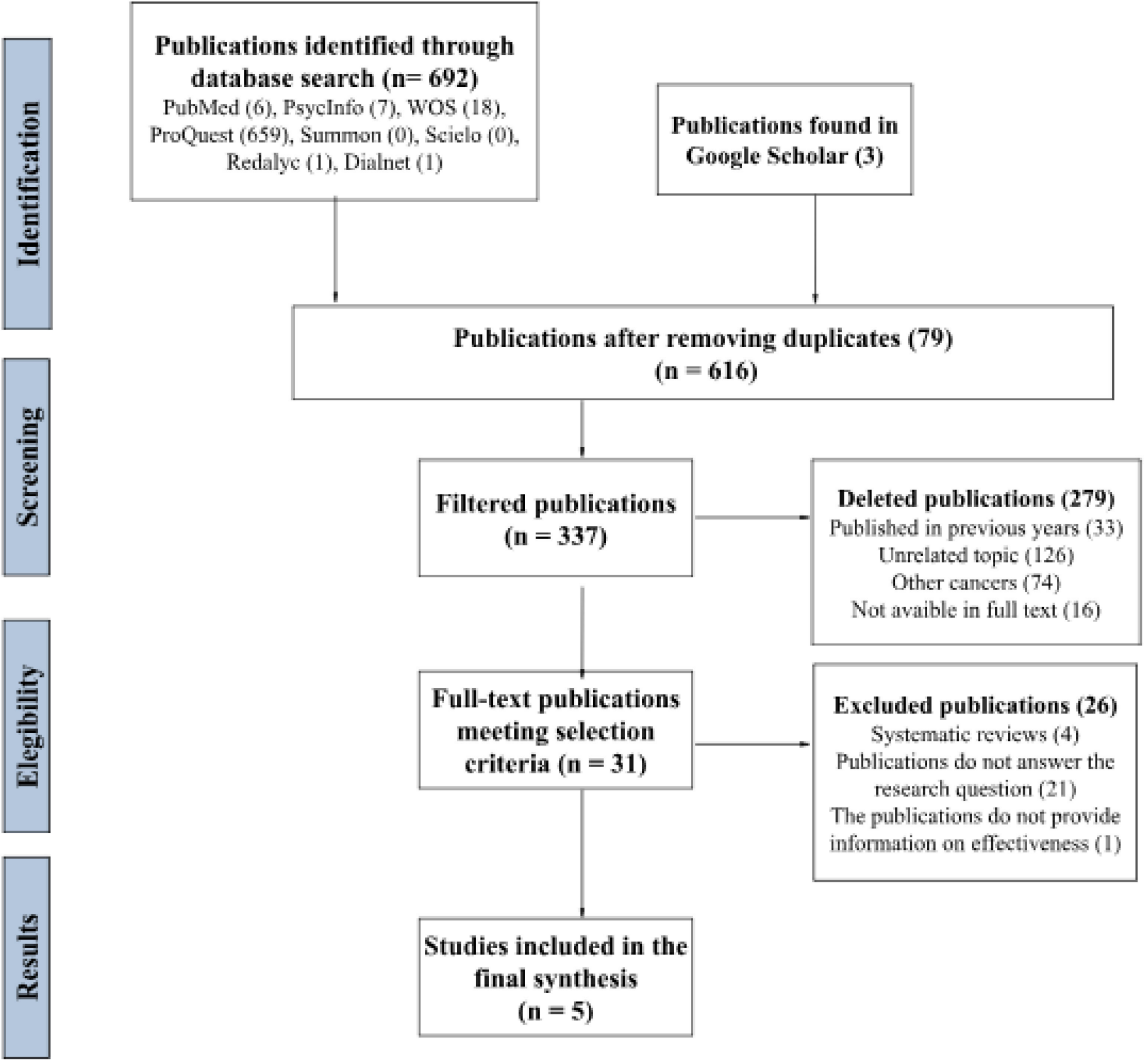
Psychological treatment. PRISMA-based systematic review process flowchart.

Two distinct selection processes were performed, each corresponding to one of the research questions: (a) identifying validated instruments for assessing Fear of Cancer Recurrence (FCR) in patients with hematological cancer, with a focus on reliability and validity, and (b) evaluating the most effective psychological interventions for reducing FCR in this patient population, particularly through randomized controlled trials (RCTs). This process yielded six studies for the first research question and five for the second (Figures 1, 2).

## Results

The results have been organized into two categories, corresponding to the research questions and search processes outlined in the diagrams (Figures 1, 2): (a) selected studies examining validated instruments for assessing Fear of Cancer Recurrence (FCR) in patients with hematological cancer, with a focus on reliability and validity [(Clever et al., 2018; Hinz et al., 2015, 2024; Luz et al., 2020; Park, 2024; Xu et al., 2022) see Table 4], and (b) studies reviewing the most effective psychological interventions for reducing FCR in this patient population, particularly randomized controlled trials [RCTs; Arch et al., 2024; Brooker et al., 2020; Deuning-Smit et al., 2023, 2024; Lamarche et al., 2023 (see Table 5)].

**TABLE 4:** Variables for outcome analysis.

Variable category	Description
Variables related to the sample.	The selected studies include a sample with a significant number of oncohematological patients, conducted across various age groups and consisting of participants of both genders.
Variables related to the measurement instruments.	The measurement instruments used in the study must have been previously empirically validated. Additionally, they should have the sole objective of measuring FCR (Fear of Cancer Recurrence).
Variables related to methodological quality and risk of bias.	The methodological quality analysis of the selected studies was conducted using the Robins-I scale (Arch et al., 2024) and the PEDro scale (Lamarche et al., 2023).
Variables related to the outcomes.	The articles included in the systematic review must report statistically significant results, and therefore, the effect size has been taken into account.

**TABLE 5:** Main characteristics of the studies included in the systematic review that address the question related to the assessment instruments.

Author and year	Sample	Study objective	Instruments	Results	Conclusions
Hinz et al., 2015	The sample consists of 2,059 patients, of whom 125 are hematologic patients. Age: 17–93 years. Men: 1,210 Women: 849	To evaluate the psychometric properties of the FoP-Q-12 questionnaire.	- FoP-Q-12: Measures fear of recurrence. - HADS: Measures anxiety and depression. Only the anxiety subscale is used. - GAD-2: Measures generalized anxiety. - EORTC QLQ-C30: Assesses quality of life in cancer patients.	Reliability: α = 0.90 Factor analysis: Bayesian information criterion: 1263.4 Comparative fit index: 0.883 Tucker-Lewis index: 0.857 Chi^2^: 10,421 Root mean square error: 0.050 Effect size: *r* = 0.71	The FoP-Q-12 proved to be a valid instrument for measuring fear of progression in cancer patients.
Hinz et al., 2024	The sample consists of 1,733 patients, of whom 202 are hematologic patients. Age: 18–50 years Men: 702 Women: 1,031	To test the psychometric properties of the FoP-Q-12 and CARQ-4 questionnaires, determine their relationship with other anxiety-related constructs, and analyze the impact of sociodemographic and clinical factors on Fear of Cancer Recurrence (FCR).	- FoP-Q-12: Measures fear of recurrence. - CARQ-4: Measures fear of recurrence. - GAD-7: Measures generalized anxiety. - WI-7: Measures health-related anxiety/worry. - PHQ-9: Measures depression.	Reliability: FoP-Q-12: α = 0.895 CARQ-4: α = 0.915 Correlation: *r* = 0.72	Both instruments are useful for measuring FCR. FCR levels are relatively high and largely depend on the definition of cutoff scores. Radiotherapy, chemotherapy, and antibody therapy (but not surgery) lead to increased FoP. Female patients and middle-aged patients have higher FCR.
Park, 2024	The sample consists of 93 patients, of whom 24 are hematologic patients. Age: 20–82 years Men: 17 Women: 76	To explore the psychometric properties of the FCRI-S subscale and evaluate its applicability to cancer survivors in South Korea.	- FCRI-S: Assesses the intrusion and severity of thoughts related to FCR. - CWS: Measures FCR. - FoP-Q-SF: Measures FCR. - PHQ-9: Measures depressive symptoms. - MOS-SSS: Measures perceived social support.	Reliability: α = 0.88 Validity: Convergent validity: Strong correlation with CWS (*r* = 0.80) and FoP-Q-SF (*r* = 0.69). Discriminant validity: Weaker correlations with PHQ-9 (*r* = 0.37) and MOS-SSS (*r* = −0.23).	The FCRI-S subscale demonstrates good internal consistency and construct validity among South Korean cancer survivors. This instrument can be effectively used to detect and assess FCR in clinical settings.
Luz et al., 2020	La muestra se compone de 32 pacientes de los cuales 23 son pacientes hematológicos. Edad: 10–18 años. Niños: 15 Niñas:17	Adaptar la versión adulta de FoP-Q-SF para niños y examinar las propiedades psicométricas en pacientes de cáncer pediátrico.	-FoP-Q-SF: mide FCR. -DIKJ: mide síntomas depresivos. -Tres escalas (fobia a los animales, ansiedad al tratamiento médico, ansiedad por separación) del PHOKI: mide ansiedad y miedos diferentes al FCR. -ILK: mide calidad de vida. -FEEL-KJ: explora estrategias de afrontamiento adaptativas para la regulación de la ansiedad.	Fiabilidad α = 0,86 Validez convergente: con depresión (*r* = 0.52), con fobia animal (*r* = 0.33), con ansiedad por separación (rs = 0.62), con procedimientos médicos (rs = 0.76). Validez divergente: FEEL-KJ (*r* = 0.41)	El cuestionario adaptado FoP-Q-SF/C se puede utilizar para investigar FCR en pacientes pediátricos con cáncer. El FCR es un constructo que ocurre en niños y adolescentes con cáncer. Sugiere más investigaciones con una muestra más grande para validar y estandarizar el FoP-Q-SF/C.
Clever et al., 2018	The sample consists of 32 patients, of whom 23 are hematologic patients. Age: 10–18 years Boys: 15 Girls: 17	To adapt the adult version of the FoP-Q-SF for children and examine its psychometric properties in pediatric cancer patients.	- FoP-Q-SF: Measures FCR. - DIKJ: Measures depressive symptoms. - Three scales from PHOKI (animal phobia, medical treatment anxiety, separation anxiety): Measure anxiety and fears unrelated to FCR. - ILK: Measures quality of life. - FEEL-KJ: Explores adaptive coping strategies for anxiety regulation.	Reliability: α = 0.86 Validity: Convergent validity: Correlation with depression (*r* = 0.52), animal phobia (*r* = 0.33), separation anxiety (rs = 0.62), and medical procedures anxiety (rs = 0.76). Divergent validity: Correlation with FEEL-KJ (*r* = 0.41).	The adapted FoP-Q-SF/C questionnaire can be used to assess FCR in pediatric cancer patients. FCR is a relevant construct in children and adolescents with cancer. Further research with a larger sample is recommended to validate and standardize the FoP-Q-SF/C.
Xu et al., 2022	326 oncohematologic patients Age: 25–79 years Men: 145 Women: 181	To evaluate the psychometric properties of the Chinese Fear of Cancer Recurrence Inventory (FCRI-C) in follicular lymphoma survivors.	- FCRI: Measures FCR. - EORTC QLQ-C30: Assesses quality of life in cancer patients. - EQ-5D-5L: Measures quality of life. - PHQ-9: Assesses depressive symptoms. - MOS-SSS: Measures perceived social support.	Internal consistency: Intraclass correlation coefficient = 0.82, α = 0.95. Factor analysis: RMSE = 0.063, CFI = 0.92 (bifactor model). Convergent validity: Correlations with QLQ-C30 (*r* = 0.31) and EQ-5D (*r* = 0.41).	The FCRI-C is a valid and reliable instrument for measuring and detecting FCR levels in Chinese survivors of follicular lymphoma.

The analysis of these primary studies was guided by key variables, as detailed in Table 3.

### Variables related to the sample

A total of 800 patients diagnosed with hematologic cancer participated in the selected studies, with the overall sample comprising 4,515 oncology patients. Most studies included participants of both sexes (Figure 1), with the sole exception of Lamarche et al. (2023), whose sample consisted exclusively of women.

Clever et al. (2018) conducted their study on a sample of parents of pediatric patients, providing data from both patients and their caregivers. Regarding age distribution, all studies reported age-related data. However, Clever et al. (2018) only provided the mean age of the pediatric patients while specifying age ranges for the parent sample.

With respect to control group inclusion, only one study (Brooker et al., 2020) incorporated a control group. However, it did not provide details on its composition in terms of age or sex.

### Variables related to the measurement instruments

The studies examining assessment instruments (Clever et al., 2018; Hinz et al., 2015, 2024; Luz et al., 2020; Park, 2024; Xu et al., 2022) provide a comprehensive evaluation of their applicability and psychometric properties. The following questionnaires are described:

- *Fear of progression questionnaire FoP-Q-12*: The Fear of Progression Questionnaire-12 (FoP-Q-12; Mehnert et al., 2006) is a shortened, 12-item version of the original 43-item Fear of Progression Questionnaire (FoP-Q; Herschbach et al., 2005). This instrument has been employed in three of the six studies reviewed (Hinz et al., 2015, 2024).

- *Fear of Progression Questionnaire for parents of children with cancer (FoP-Q-SF/PR)*: An adapted version of the FoP-Q-12 was developed to assess fear of progression in parents of children with cancer, involving the reformulation of its 12 items. The four scales were accordingly modified to reflect key domains: affective reactions, family, school, and loss of autonomy (Schepper et al., 2015). This adapted questionnaire was utilized in one of the reviewed studies (Clever et al., 2018).

- *Fear of Progression Questionnaire for Children (FoP-Q-SF/C)*: One of the studies aims to adapt the FoP-Q-SF for use with children. This adaptation was carried out by reformulating the items from the perspective of children and adolescents (Luz et al., 2020).

- *Fear of Cancer Recurrence Inventory (FCRI)*: It is composed of seven domains: triggers, severity, psychological distress, functional impairments, knowledge, reassurances, and coping strategies (Simard and Savard, 2009). Two studies utilize this questionnaire (Park, 2024; Xu et al., 2022). In the case of Park (2024), the focus is solely on the severity subscale (FCRI-S). A comparative analysis revealed that the FCRI offers a multidimensional assessment including cognitive, behavioral, and emotional components of FCR, whereas the FoP-Q-12 provides a shorter, more pragmatic measure suitable for clinical use. Both instruments demonstrated high internal consistency (α = 0.86–0.95), but only the FCRI has shown preliminary evidence of sensitivity to change in intervention studies. These distinctions highlight the complementary value of both tools depending on study objectives and clinical context.

- *Concerns about Recurrence Questionnaire (CARQ-4)*: It is a 4-item scale, three of which are derived from the Fear of Cancer Recurrence Questionnaire. Total scores range from 0 to 40, with scores of 12 or higher indicating elevated levels of FCR. This questionnaire is used in one study (Hinz et al., 2024).

### Variables related to the outcomes

The results presented below are categorized according to the objective of each study (Tables 5, 6), taking into account the effect size when provided, as well as other psychometric properties.

**TABLE 6:** Main characteristics of the studies included in the systematic review that address the question related to the treatments.

Author and year	Sample	Objective of the study Treatment/program^1^	Instruments	Results	Conclusions
Brooker et al., 2020	Of the 27 participants, 10 were hematologic patients. Age: 38–84 years. Men: 10 Women: 17 Control group: 127 patients.	To examine the feasibility and acceptability of an adaptation of the Mindful Self-Compassion (MSC) program among adult cancer patients. To assess pre- and post-program changes in psychosocial well-being.	- DASS-21: Used to assess symptoms of depression and stress experienced over the past week. - FCRI-SF: Measures the severity of fear of cancer recurrence. - UCLA Loneliness Scale Version 3: Measures loneliness. - BAS: Assesses body image. - CAMS-R: Measures the level of mindfulness. - SCS: Assesses self-compassion.	Effect size: FCR (*r* = 0.47), depression (*r* = 0.50), stress (*r* = 0.43), loneliness (*r* = 0.43), body image satisfaction (*r* = 0.22), mindfulness (*r* = 0.57), and self-compassion (*r* = 0.37).	The MSC program appears to be feasible and acceptable for adults diagnosed with non-advanced cancer. Participation in the program was associated with improvements in psychosocial well-being (with a substantial effect size in FCR).
Deuning-Smit et al., 2023	Of the 19 participating patients, 2 were hematologic patients. Age: 25–73 years. Men: 5 Women: 14	To identify barriers and facilitators for implementing the evidence-based combined SWORD intervention in routine psycho-oncological care.	- CWS-6: Measures Fear of Cancer Recurrence (FCR).	The data were coded following a thematic analysis based on the theoretical framework of Grol and Flottorp. Six domains were described: (1) innovation, (2) professionals, (3) patients, (4) social context, (5) organization, and (6) economic and political context.	Implementation strategies should target the patient, professional, organizational, and economic and political domains. The identified barriers and facilitators are relevant to other psycho-oncology researchers aiming to bridge the gap between research and practice. This study contributes to the implementation of evidence-based psychological interventions for cancer patients.
Arch et al., 2024	The sample consists of 29 patients, of which 5 are hematologic patients. Age: 42–75 Women: 19 Men: 10	The study aims to develop and test a written exposure coping intervention (EASE) focused on the worst-case scenario among adults with advanced cancer who report trauma symptoms or elevated Fear of Progression (FoP) related to cancer.	- IES-R: Measures the impact of events (cancer). - FoPQ-S and CARS: Measure fear of progression. - CRS: Measures fear of cancer recurrence (FCR). - GAD-7: Measures anxiety symptoms. - PHQ-8: Measures depression symptoms. - HAIQ: Measures hopelessness. - DAPR: Measures fear of death. - PROMIS-SF: Assesses pain and fatigue interference. - COPE: Evaluates coping strategies. - VQ: Assesses values. - FFMQ: Measures mindfulness skills. - SCS-SF: Measures self-compassion.	Effect size for FoP: *d* = −0.95 (CARS) *d* = −0.77 (FoPQ-S).	EASE is a promising approach to reduce FoP and trauma-related symptoms in advanced cancer adults, justifying further studies.
Lamarche et al., 2023	The sample consists of 10 family members of patients, 4 of whom are hematologic patients. Age: 32–63 years. Men: 0 Women: 10	The study aims to adapt the in-person FORT therapy method for family caregivers and test its usability when offered in a virtual format.	- FCRI-SF: Measures fear of cancer recurrence (FCR).	Qualitative analysis: Four key themes were identified: Usability of FC-FORT. Satisfaction and engagement with content. Group cohesion. Impact of FC-FORT. No quantitative data was provided.	This study demonstrated the usability of the adapted FC-FORT with family caregivers in a virtual format.
Deuning-Smit et al., 2024	The sample consists of 59 patients, 7 of whom are hematologic patients. Age: 22–74 years. Men: 8 Women: 51	The study aims to investigate the feasibility of implementing the SWORD program in real-world psycho-oncological practice.	- CWS-6: Measures fear of cancer recurrence (FCR). - FCRI-SF: Measures fear of cancer recurrence (FCR). - EORTC-C30: Measures quality of life in cancer. - ACES: Measures expectations of anxiety change.	Effect size: *p* < 0.001, ηp^2^ = 0.694	SWORD proves effective in reducing FCR when applied in a real-world care setting.

Treatment/program: - Mindful Self-Compassion (MSC) Program: This is a manualized program taught by certified instructors (Germer and Neff, 2019; Center for Mindful Self-Compassion, 2017). It is an approach designed to help individuals increase their resilience through mindfulness and self-compassion. To reduce the burden on patients, one of the selected studies shortens the session duration from 150 to 105 min and omits the 4-h retreat midway through the program. - Survivors’ Worries of Recurrent Disease intervention (SWORD): Specifically designed to address fear of recurrence, it is based on cognitive-behavioral therapy, combining stress control strategies and coping skills. - Fear Of Recurrence Therapy (FORT): This therapy was specifically designed to treat FCR from a cognitive-existential perspective. One of the studies proposes an adaptation to treat family caregivers (FC-FORT). - Written Exposure Coping Therapy (EASE): Advanced cancer survivors participate in written exposure to the worst imagined future scenario with cancer. EASE adapts written exposure therapy to help advanced cancer survivors with high levels of FOP or trauma-related symptoms reduce their fear of the future. Written exposure therapy is a brief treatment designed to address post-traumatic stress. This therapy involves only the imagined presence of the disturbing object or experience. The therapist guides the person to recall the traumatic experience and write in detail the thoughts and emotions they had at the time of the event.

*Measurement of FCR (Fear of Cancer Recurrence)*: All the questionnaires examined in these studies have demonstrated good reliability and validity. The FoP-Q-12 maintains strong reliability indices in the two studies where it was evaluated, with α = 0.90 in Hinz et al. (2015) and α = 0.89 in Hinz et al. (2024). Similarly, the child-adapted version (FoP-Q-SF) shows strong properties in both reliability and validity in the study by Luz et al. (2020). Likewise, the parent-adapted version, FoP-Q-SF/PR, displays good psychometric properties according to Clever et al. (2018). The results for the CARQ-4 are even more favorable, with an α = 0.915. Additionally, the FCRI-S yields good results for both reliability (α = 0.88) and convergent and discriminant validity (Park, 2024). The Chinese version (FCRI-C) also performs well, with an α = 0.95, as reported by Xu et al. (2022).

*Reduction of FCR (Fear of Cancer Recurrence) levels:* Participation in MSC therapy is associated with a substantial effect size for FCR, as reflected in the study by Brooker et al. (2020). Regarding the SWORD therapy presented by Deuning-Smit et al. (2023) and Deuning-Smit et al. (2024), it can be asserted that it is effective in reducing FCR, as evidenced by a large effect size reported in their latest work. The EASE therapy also demonstrates a significant effect size, measured with two questionnaires (*d* = −0.95 on CARS and *d* = −0.77 on FoPQ-S). Lamarche et al. (2023) suggest that the FC-FORT therapy is beneficial for caregivers; however, they do not provide data related to the effect size. The reviewed interventions varied in length from 4 to 10 sessions and were delivered in face-to-face, blended, or online formats. Mindful Self-Compassion (MSC) and SWORD therapies demonstrated medium-to-large effect sizes (Cohen’s *d* = 0.60–0.85) in reducing FCR in mixed oncology samples. EASE and FC-FORT, while promising, remain supported mainly by pilot data. Table 7 a summary of intervention characteristics, delivery modes, and observed effect magnitudes.

**TABLE 7:** Intervention characteristics, delivery modes, and observed effect magnitudes.

Author and year		Intervention characteristics, delivery modes, and observed effect magnitudes			
Hinz et al., 2015		FoP-Q-12 questionnaire evaluated; strong reliability (α = 0.90).			
Hinz et al., 2024		FoP-Q-12 questionnaire evaluated; strong reliability (α = 0.89).			
Park, 2024		FCRI-S evaluated; reliability (α = 0.88) and good convergent and discriminant validity.			
Luz et al., 2020		FoP-Q-SF (child-adapted version) evaluated; strong reliability and validity.			
Clever et al., 2018		FoP-Q-SF/PR (parent-adapted version) evaluated; good psychometric properties.			
Xu et al., 2022		FCRI-C (Chinese version) evaluated; good reliability (α = 0.95).			
Brooker et al., 2020		Participation in MSC therapy associated with a substantial effect size for FCR reduction.			
Deuning-Smit et al., 2023		SWORD therapy effective in reducing FCR.			
Lamarche et al., 2023		FC-FORT therapy beneficial for caregivers; effect size not reported.			
Deuning-Smit et al., 2024		SWORD therapy effective; large effect size reported.			
**Author and year**	**Reference**	**Design / focus**	**FCR/FoP measure**	**Effect size (value)**	**Key notes**
Brooker et al., 2020	Brooker et al., 2020. Adaptation of Mindful Self-Compassion (MSC) in adult cancer patients (Palliative and supportive care, 18:130–140)	Pre-post feasibility study (no control group)	FCR/FoP (Fear of cancer recurrence/ progression)	***r* = 0.47** (pre-post, Wilcoxon)	The results table reports pre-post changes and effect sizes for several variables; for FCR/FoP they report *r* (not *d*). Also: Depression ***d* = 0.50**, Stress ***d* = 0.43**, Mindfulness ***d* = 0.57**, etc.
Deuning-Smit et al., 2023	Deuning-Smit et al., 2023. Barriers and facilitators for implementation of SWORD (J Cancer Surviv, 17:1057–1071)	Qualitative (interviews) on implementation	–	Not applicable (no intervention effects)	Identifies implementation barriers and facilitators; no effect sizes reported because efficacy was not evaluated.
Arch et al., 2024	Arch et al., 2024. Written exposure to worst-case scenarios (EASE) in advanced cancer (J Palliat Med)	Pre-post pilot intervention	FoP and trauma symptoms	“Predominantly large effect sizes” (no values reported)	The abstract describes mostly large effects but does not provide numeric *d/g/r*-values; full text is restricted.
Lamarche et al., 2023	Lamarche et al., 2023. Virtual FC-FORT for family caregivers (Front Digit Health, 5:1129536)	Usability/adaptation study (not efficacy)	FCR in caregivers	Notreported (within this study)	Evaluates usability; cites prior FORT efficacy results but does not estimate effect sizes for this virtual version.
Deuning-Smit et al., 2024	Deuning-Smit et al., 2024. Toward implementation of an evidence-based intervention (SWORD) (Psycho-Oncology, 33:e6297)	Feasibilityin real-world psycho-oncology practice	FCR (SWORD implementation)	Not reported	Examines feasibility and implementation outcomes; the abstract does not provide effect sizes.

Bold values indicate statistically significant results (*p* < 0.05). Effect sizes are reported as r for pre-post changes and as d when specified in the original studies.

### Variables related to methodological quality and risk of bias

**FIGURE 3:**
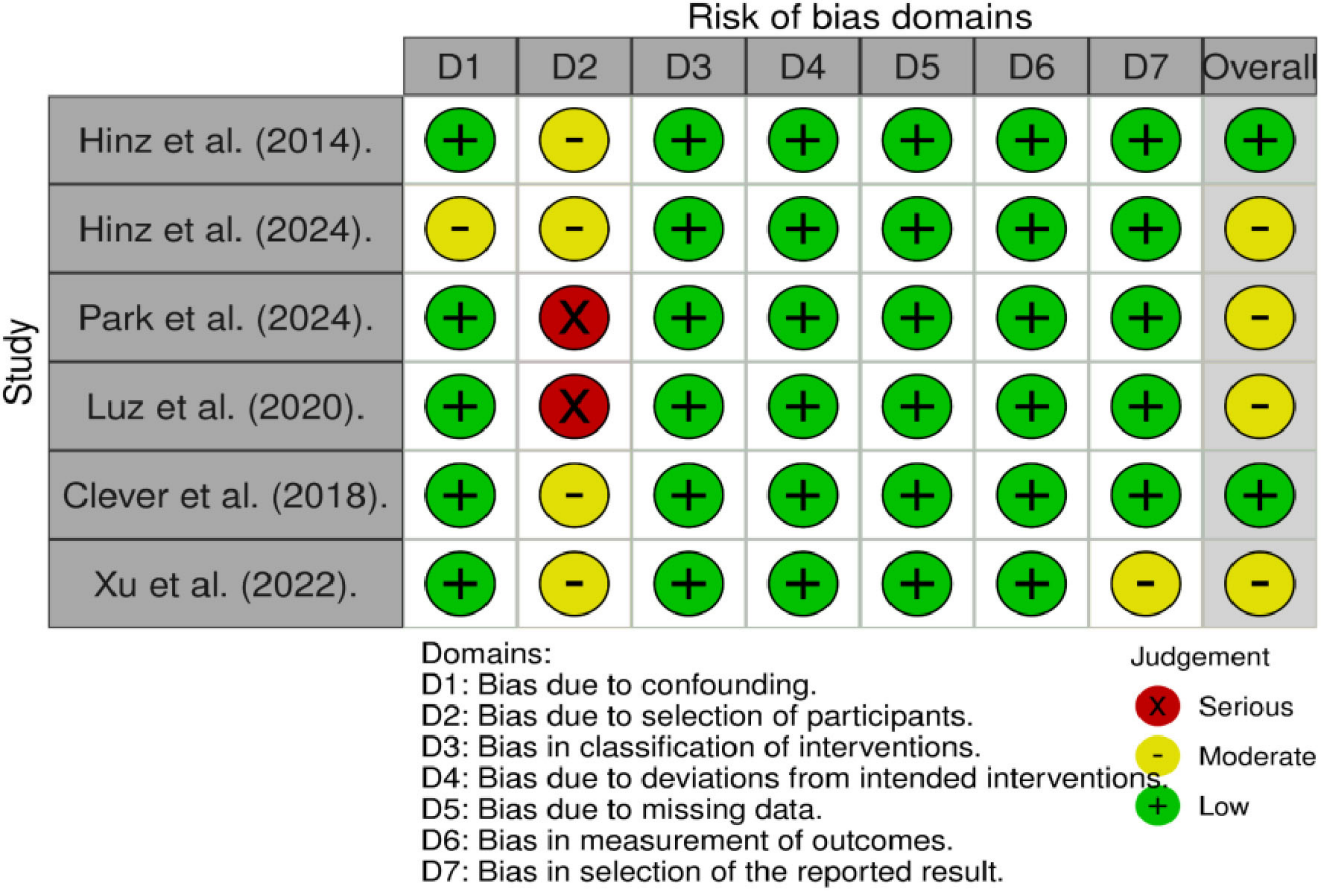
Results of the ROBINS-I scale. 1. Confounding bias, 2. Participant bias, 3. Classification bias, 4. Deviation bias, 5. Bias due to missing data, 6. Outcome measurement bias, and 7. Outcome selection bias are all potential sources of bias that can affect the methodological quality of studies, influencing the validity and reliability of the results. Each type of bias can introduce systematic errors in the design, implementation, or analysis of the research, potentially distorting the findings and leading to misleading conclusions.

The methodological quality of the studies selected to address the question related to the instruments was assessed using the ROBINS-I scale (Sterne et al., 2016) for non-randomized studies (Figure 3). Specifically, a serious risk was identified concerning participant selection in two of the studies (Luz et al., 2020; Park, 2024). From an overall analysis, it is considered that four of the studies exhibit moderate bias (Sancho et al., 2024; Hinz et al., 2015; Page et al., 2021; Park, 2024). The studies selected to address the question related to treatments (Arch et al., 2024; Brooker et al., 2020; Deuning-Smit et al., 2023, 2024; Lamarche et al., 2023) were evaluated using the PEDro scale (Maher et al., 2003). The overall assessment of these studies indicates poor methodological quality in all cases, except for the study by Brooker et al. (2020), which was rated as having medium quality.

**FIGURE 4:**
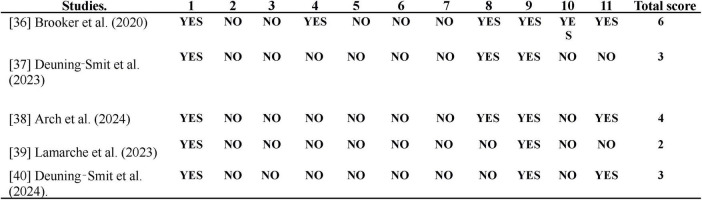
Results of the PEDro scale. 1. The selection criteria were specified; 2. Subjects were randomly assigned to groups; 3. The allocation was concealed; 4. The groups were similar at baseline regarding the most important prognostic indicators; 5. All subjects were blinded; 6. All therapists administering the therapy were blinded; 7. All evaluators who measured at least one key outcome were blinded; 8. Measures of at least one key outcome were obtained from more than 85% of the subjects initially assigned to the groups; 9. Results were presented for all subjects who received treatment or were assigned to the control group, or when this was not possible, data for at least one key outcome were analyzed by “intention to treat”; 10. Statistical comparison results between groups were reported for at least one key outcome; 11. The study provides point estimates and variability measures for at least one key outcome.

As shown in Figure 4, although all the studies describe the source of participant recruitment and inclusion criteria, none of them involved random assignment or distribution of participants. Furthermore, no study implemented blinding for participants, therapists, and/or assessors. Most studies report key outcome measures obtained from over 85% of the participants, except for the studies by Deuning-Smit et al. (2024), where the completion rate was 73%, and Lamarche et al. (2023), where only 75% of the initially recruited participants completed the therapy. Additionally, all studies presented results for all participants who received treatment. Only the study by Brooker et al. (2020) reports statistical comparisons between groups, as it is the only study to include a control group, assuming both groups are similar in terms of prognosis. Two articles (Deuning-Smit et al., 2023; Lamarche et al., 2023) do not provide data related to effect size.

## Discussion

This systematic review aimed to achieve two primary objectives: first, to identify validated instruments for assessing cancer recurrence fear (FCR) in patients with oncohematological diagnoses and to analyze their psychometric properties; and second, to evaluate the efficacy of psychological interventions designed to reduce this fear in this population. Across the included studies, several widely used instruments were identified, notably the Fear of Progression Questionnaire (FoP-Q-12) and the Fear of Cancer Recurrence Inventory (FCRI), both demostrating strong psychometric properties. However, a critical examination of methodological limitations and applicability to the hematologic population remains essential.

Although the reviewed questionnaires generally exhibit satisfactory reliability and validity in mixed oncological samples, only one study (Xu et al., 2022) employed an exclusive oncohematological cohort. This limitation is significant, as the clinical and emotional profiles of hematologic cancer o patients may differ substantially from those with other types of cancer. The heterogeneity of study samples complicates the generalization of findings, underscoring the need for future research that specifically evaluates psychometric performance within hematologic populations. Furthermore, most included studies combined solid and hematologic tumor samples, further restricting generalizability. The predominance of quasi-experimental and pre–post designs without control groups also constrains causal interpretation. These methodological shortcomings highlight the need for robust randomized controlled trials (RCTs) with larger, diagnosis-specific cohorts.

Conceptual clarity also emerged as a critical issue. Several studies used the constructs fear of cancer recurrence (FCR) and fear of cancer progression (FoP) interchangeably, despite previous evidence (Coutts-Bain et al., 2022) indicating that they represent related but distinct phenomena. This conceptual overlap may compromise the interpretation and comparability of findings, reinforcing the need for greater rigor and definitional precision in future research.

Regarding psychological interventions aimed at reducing FCR, the review revealed a scarcity of rigorously designed studies. Among the selected works, only Brooker et al. (2020) included a control group, limiting the strength of conclusions about therapeutic efficacy. The absence of RCTs and the lack of long-term follow-up assessments further hinder the determination of sustained effects. Moreover, most interventions were applied to mixed oncological samples, raising questions about their specific applicability to hematologic patients. While some interventions, such as Mindful Self-Compassion (MSC; Brooker et al., 2020) and SWORD therapy (Deuning-Smit et al., 2023, 2024), demonstrated large effect sizes, the absence of data focusing exclusively on hematologic patients necessitates cautious interpretation. Similarly, Lamarche et al. (2023) suggested benefits of FC-FORT for caregivers, but the lack of quantitative data precludes firm conclusions regarding its efficacy.

The reviewed interventions also show differential alignment with the psychological profiles and unmet needs of hematologic cancer survivors. SWORD, a blended cognitive-behavioral therapy, addresses worry, avoidance, and catastrophic thinking—mechanisms particularly prevalent among patients facing long-term treatment uncertainty. Mindful Self-Compassion (MSC) interventions may be especially beneficial for survivors exhibiting high levels of self-criticism, shame, or guilt—transdiagnostic factors closely linked to FCR. EASE therapy, emphasizing emotional engagement and symptom acceptance, appears suitable for individuals prone to emotional suppression or avoidance. Finally, FC-FORT, developed for caregivers, targets dyadic distress and shared recurrence fears—relevant features in hematologic contexts where caregiver FCR often parallels patient distress. These therapy–profile matches support the rationale for personalized approaches within oncohematology.

Future research should aim to integrate biomarker and physiological data into psychological assessment frameworks to enhance personalization and treatment precision. Potential indicators include stress-related biomarkers (e.g., diurnal cortisol slope, heart rate variability), sleep and actigraphy parameters, and inflammatory markers (CRP, IL-6) that reflect stress reactivity and emotional regulation. In hematologic oncology, clinical data such as minimal residual disease (MRD) status or cytokine profiles following HSCT or CAR-T therapy could inform individualized psychological care and optimal timing of interventions. This bio-behavioral integration aligns with current trends in precision psycho-oncology.

The findings from this review highlight an urgent need for research specifically addressing FCR in hematologic cancer populations. Longitudinal designs are needed to evaluate the stability of FCR over time and the durability of intervention effects. Future RCTs should incorporate methodological refinements, including stratification by diagnosis and treatment modality (e.g., leukemia, lymphoma, HSCT, CAR-T, TKI therapy), use of active control groups, blinded assessments, and validated FCR measures (e.g., FCRI, FCRI-SF). Combining traditional measures with ecological momentary assessment or digital monitoring may further enhance accuracy. Hybrid or blended delivery formats, such as SWORD, could improve scalability and adherence, while dyadic approaches like FC-FORT can capture caregiver–patient dynamics that influence outcomes.

Stratifying FCR by treatment modality may also clarify distinct emotional adaptation trajectories. For instance, lymphoma survivors often experience prolonged surveillance and relapse uncertainty, fostering chronic vigilance, whereas leukemia patients undergoing continuous or maintenance therapy face persistent stress related to biomarker monitoring and treatment dependence. Understanding these differentiated patterns may guide tailored intervention timing and design. Recent evidence supports this perspective, revealing elevated FCR prevalence and distinct emotional correlates among lymphoma survivors (Latella et al., 2020).

Finally, sociodemographic and psychological factors should be incorporated into FCR research frameworks. Previous studies have linked FCR to quality of life (Ellis et al., 2022), generalized anxiety (Göbel et al., 2023), and social support (Hu et al., 2021), reinforcing the need for an integrative biopsychosocial approach.

## Conclusion

In conclusion, this review identifies validated tools for assessing FCR and highlights promising interventions, while also exposing critical evidence gaps. Clinically, psycho-oncologists may employ the FCRI as a screening tool to identify hematologic patients at risk of persistent FCR. Interventions such as SWORD (CBT-based, blended delivery) and MSC (compassion-focused) are feasible within hospital psycho-oncology settings, whereas FC-FORT offers a model for addressing caregiver-related FCR. Adapting these approaches to hematologic contexts could enhance emotional adjustment and dyadic coping, advancing precision in psycho-oncological care.

## Data Availability

The original contributions presented in this study are included in this article/supplementary material, further inquiries can be directed to the corresponding author.
